# Commentary: Do people really care less about their cats than about their dogs? A comparative study in three European countries

**DOI:** 10.3389/fvets.2024.1332301

**Published:** 2024-02-07

**Authors:** Catarina Jota Baptista, Fernanda Seixas, José M. Gonzalo-Orden, Paula A. Oliveira

**Affiliations:** ^1^Departamento de Ciências Veterinárias, Escola de Ciências Agrárias e Veterinárias (ECAV), Universidade de Trás-os-Montes e Alto Douro, Vila Real, Portugal; ^2^Centro de Investigação das Tecnologias Agroambientais e Biológicas (CITAB-Inov4Agro), Universidade de Trás-os-Montes e Alto Douro, Vila Real, Portugal; ^3^Instituto de Biomedicina (IBIOMED), Universidad de León, León, Spain; ^4^Egas Moniz Center for Interdisciplinary Research (CiiEM), Egas Moniz School of Health &Science, Almada, Portugal; ^5^Associated Laboratory for Animal and Veterinary Sciences (AL4AnimalS), Centro de Ciência Animal e Veterinária (CECAV), Universidade de Trás-os-Montes e Alto Douro, Vila Real, Portugal

**Keywords:** dog, cat, pet care, caregiver, letter (to the Editor)

We would like to express our scientific opinion on the paper “*Do people really care less about their cats than about their dogs? A comparative study in three European countries*,” published by Sandøe et al. 2023 (Frontiers in Veterinary Sciences—Section Veterinary Humanities and Social Sciences, Vol. 10, doi: 10.3389/fvets.2023.1237547).

We believe it is important to understand pet owners' perspectives and feelings regarding their companion animals to provide a better service as veterinary professionals (1), so studies like the one provided by these authors contribute to this crucial goal in companion animal medicine. However, from our professional perspective, not all the presented indicators or methods may reveal information about the affection and care that owners have for their animals, which is the question that we believe the authors intended to answer with their work.

A detailed analysis of the questionnaire (published as supplementary data) raises some bias concerns. The owners that accept to spend some of their time to participate in a survey regarding their pets and their relationship with them reveal, from the beginning, a concern about the topic and, consequently, about their animals. At the instructions and aims of the questionnaire, at the first page, it is possible to read “to explore why some people keep pets and others do not, and to explore the attitudes of pet owners to developments in modern small animal practice.” Owners with other interests that are not related with animals would maybe ignore the questionnaire. Moreover, even though the questionnaire is generally well-designed, some questions require personal perspective regarding the role of pets in a person's life, and it may be not related with the value that the subject attributes to their pet. As an illustration, I am not sure if assuming “I consider my pet to be a friend” or “My pet knows when I'm feeling bad” reveals that much about truly caring about the pet or how much they emotionally invest in their dog or cat. Some recent scientific evidence suggests that there is a mutual bond between pets and their owners that helps in the recognition of facial expressions and emotions, but some pet owners may not believe or know this but significantly care and value their pets (2, 3). Historically, the domestication of dogs required frequent human-dog (heterospecific) communication and understanding, while the domestication and relationship with cats was more commensal and independent, typically hunting rodents (3–5). But none of these are synonyms of caring or affection, specially nowadays. Some owners are almost incredibly skeptical about animals' feelings, emotions, complex rational thinking, among other topic of animal psychology and behavior sciences, but still recognize a remarkable importance of dogs or cats in their life. In other words, animal care and affection must be clearly distinguishable of anthropomorphism (6).

Furthermore, we must mention that the possession of pet health insurance may not as well reveal that much about caring. In most cases, dogs (especially large breeds) are much more expensive during their lifetime than a cat or a small breed dog, considering their nutrition needs but also regarding veterinary services, i.e., they normally require higher doses of drugs (as more tablets) or more expensive surgeries due to their complexity or higher doses of anesthetics. Possibly, in most countries, the acquisition of pet health insurance may be advantageous and appealing for the owners of dogs (especially large dogs) than cats, without meaning that the first group is more important for their owners. On the other hand, the veterinary costs associated with dogs' treatment are often more expensive, comparing to cats. In other words, the decision of acquiring a pet health insurance policy can simply be based on a better economic option. Furthermore, the options and the generalization of animal health insurance will, of course, depend very much on the countries and the offer made available by the insurance companies.

In conclusion, “*Do people really care less about their cats than about their dogs? A comparative study in three European countries*” provides a general idea about the behavior and attitude of the pet owners of dogs and cats and contributes to improve a different approach of cat owners and dog owners by the veterinarians. However, some bias may exist when trying to include animal owners in these studies and, furthermore, some of the indicators evaluated may be influenced by multiple factors (economic, social, psychological) and may not directly answer the objective question.

## Author contributions

CJB: Conceptualization, Writing – original draft. FS: Writing – review & editing. JMG-O: Writing – review & editing. PAO: Writing – review & editing.
